# Temporal trends and epidemiological patterns of Lassa fever outbreaks in Ebonyi State, Nigeria: A retrospective study (2018–2023)

**DOI:** 10.1371/journal.pgph.0006871

**Published:** 2026-07-28

**Authors:** Paul M. Iziomo, Emmanuel Awosanya, Olalekan John Taiwo, Judah Moyin-Jesu, Gift Idumah, Samuel Ayanwale, Eniola Cadmus, Terese Gabriel Orum, Ogbonna Nwambeke, Victor Akinseye, Oyewale Tomori, Simeon Cadmus

**Affiliations:** 1 Damien Foundation Centre for Genomics and Global Health, University of Ibadan, Ibadan, Nigeria; 2 Department of Veterinary Public Health and Preventive Medicine, University of Ibadan, Ibadan, Nigeria; 3 Department of Geography, University of Ibadan, Ibadan, Nigeria; 4 Department of Community Medicine, College of Medicine, University of Ibadan, Ibadan, Nigeria; 5 Regional Disease Surveillance System Enhancement Project, Ministry of Health, Abuja, Nigeria; 6 Ebonyi State Ministry of Health, Centenary City, Abakaliki, Ebonyi State, Nigeria; 7 Department of Microbiology, Nigerian Institute of Medical Research, Yaba, Lagos, Nigeria; 8 Institute of Genomics and Global Health, Redeemer’s University, Ede, Osun State, Nigeria; 9 Centre for Control and Prevention of Zoonoses, Faculty of Veterinary Medicine, University of Ibadan, Ibadan, Nigeria; Nasarawa State University, NIGERIA

## Abstract

Ebonyi State in southeastern Nigeria has experienced a notably high number of deaths from Lassa fever (LF). Despite this, there is a dearth of studies on the epidemiological and management outcomes of LF outbreaks over the years. The study aims to describe and analyze the epidemiological and management factors of LF for a period of six years (2018–2023) in Ebonyi State with a view to informing targeted interventions, control, and prevention of LF in the state. Secondary data of the LF outbreak from January 2018 to December 2023 were obtained from the Ebonyi State Ministry of Health. The primary outcome was survival from LF, assessed by identifying predictors through logistic regression and estimating survival probability using survival analysis. Associations between demographic and clinical variables were evaluated using odds ratios, chi-square, and Fisher’s exact tests. Geospatial mapping with ArcGIS explored the spatial distribution of LF cases. A total of 1,910 suspected cases were reported, with 368 confirmed. The case fatality rate (CFR)among confirmed cases was 33.97%, increasing yearly. Nearly half (49.7%) were from Abakaliki Local Government Area (LGA), showing a clustered spatial pattern. The median age was 32 years (IQR: 22–42), with 52.7% male. Dehydration (OR: 0.45), nausea/vomiting (OR: 0.57), neurologic disorders (OR: 0.33), bleeding (OR: 0.48), sepsis (OR: 0.39), and respiratory disorders (OR: 0.44) significantly reduced survival odds. Median time from symptom onset to hospital presentation was 10 days (IQR: 7–18), and from presentation to diagnosis was 12 days (IQR: 4–23). The CFR of LF in Ebonyi State is high, with Abakaliki LGA as the hotspot. Delays in hospital presentation and diagnosis exist. Regular community sensitization and improved training of medical personnel are needed to promote early symptom reporting and enhance timely LF management.

## Introduction

Lassa fever (LF) is an acute viral hemorrhagic disease caused by the *Mammarenavirus lassaense* (LASV). It is mainly transmitted to humans through contact with the urine or faeces of infected *Mastomys* rats [1]. Transmission also occurs through cuts and inhalation of virus-laden dust particles [2]. Human-to-human transmission occurs through contact with infected body fluids [3]. In mild to moderate cases, signs and symptoms may include fever, overall body weakness, malaise, headache, sore throat, muscle pain, and chest pain [4], while bleeding, nervous system, and urinary complications occur in severe cases [5]. The incubation period ranges from 1 to 3 weeks [6].

Infection with LASV poses a significant challenge in West Africa, infecting approximately 300,000 individuals and contributing to over 5,000 deaths yearly [7]. It was first identified in Nigeria in 1969 [8], and numerous cases have been reported thereafter. Most LF cases in Nigeria are reported during the dry season [9]. In 2023, Nigeria experienced a large outbreak of LF with 9155 suspected cases, five probable cases, 1270 confirmed cases and 227 deaths, resulting in a case fatality rate (CFR) of 17.9% [10]. These figures are higher than the 8297 suspected cases, 1067 confirmed cases and 7.7% CFR reported in 2022 [11]. This suggests a continued upward trend in LF activity in recent years in Nigeria. Ebonyi State, which contributed 4.4% to the total confirmed LF cases in 2022 [11], is one of the states in Nigeria that has recorded poor indices in LF management. Based on the Nigeria Centre for Disease Control and Prevention (NCDC) annual LF reports (2021–2023), Ebonyi State recorded a substantially higher average CFR (57.9%) compared with Edo (11.3%) and Ondo (15.8%) [10–12]. A study reported a 23.4% CFR among LF cases from January to May 6 of 2018 in Ebonyi State compared to the 14.6% recorded in Edo State for the same time period [13]. Despite the high CFR being reported from Ebonyi State, there are limited studies that have investigated the epidemiological and management factors influencing the high LF fatality rate in Ebonyi State. In this study, we set out to understand the epidemiological factors and management of LF for the six years (2018-2023) in Ebonyi State to inform targeted interventions, control, and prevention of LF in the state.

## Methodology

### Study area

Ebonyi State, situated in southeastern Nigeria, was created in 1996 and lies between the latitudes 5°40’ and 6°45’ North and longitudes 7°30’ and 8°46’ East [14]. It is bordered to the North by Benue State, Enugu State to the West, Imo and Abia states to the south, and Cross River State to the East. It has 13 Local Government Areas (LGAs) and an estimated population of 3.2 million people [15]. Agriculture is the primary occupation in the state [15]. The predominant seasons experienced in the state are the rainy and dry seasons [16]. Only four – Abakaliki, Afikpo North, Ebonyi, and Ezza South– of its 13 LGAs are designated as urban areas.

There are 556 public and private health facilities in Ebonyi State, comprising 13 general hospitals, 6 mission hospitals, 417 primary health centres, and 119 private hospitals/clinics [17]. The Alex Ekwueme Federal University Teaching Hospital Abakaliki (AEFUTHA) is a tertiary health care facility located in Abakaliki, and it serves as a referral facility within and outside the state [18]. The facility consists of a 27-bed isolation ward, with laboratory backup and support for the treatment of LF cases.

### Study design

A retrospective descriptive and analytical study of laboratory-confirmed LF cases reported in Ebonyi State from January 2018 to December 2023.

### Data collection

Records of 1910 suspected LF cases, comprising of 1,425 laboratory-negative cases, 117 pending cases awaiting laboratory confirmation at the time of data extraction, and 368 laboratory-confirmed cases were obtained from the Surveillance Outbreak Response Management and Analysis System (SORMAS) platform through the Ebonyi State Ministry of Health on January 4, 2024. SORMAS is the national disease surveillance information system designed by researchers and public health experts from Nigeria and Germany during the 2014–2015 Ebola outbreak in West Africa [19]. It receives real-time reports of diseases like Ebola, Mpox, LF, and COVID-19 from all levels of the health system, including primary, secondary, and tertiary health facilities. Suspected LF cases are entered into SORMAS when they meet the NCDC standard case definition [5], at presentation or at any point during admission if symptoms evolve. The SORMAS-based surveillance system has been reported to have a remarkable 100% sensitivity in detecting laboratory-confirmed cases, and a positive predictive value of 42%, suggesting potential overreporting of suspected cases [20].

### Case definition and record classification

Suspected cases were patients presenting to health facilities and those identified in communities during active case search, with symptoms consistent with the NCDC suspected case definition [5]. For each suspected case, clinicians and surveillance officers apply the NCDC definitions based on clinical symptoms and epidemiological risk factors. During the study period, the NCDC LF case definitions remained consistent and were uniformly applied within SORMAS. Confirmed cases were defined as suspected or probable cases with laboratory confirmation by polymerase chain reaction. Pending cases were suspected cases awaiting laboratory confirmation. Negative cases were suspected cases with negative laboratory results. This analysis was restricted to laboratory-confirmed cases, while suspected, pending, negative, and “not a case” records were excluded from epidemiological, outcome, and spatial analyses to reduce misclassification and ensure diagnostic certainty. All confirmed cases were admitted and managed at AEFUTHA, the designated LF treatment centre for the state. Case outcome was defined as the status of the case, either alive or dead, at the time of exit to the hospital. Some symptoms were combined into a single variable due to their tendency to be interconnected. The categorization is summarized in the table below (Table 1).

**Table 1:** Symptoms categorization.

S/N	Symptoms	Variable
1.	Lack of appetite, anorexia and refusal to eat or drink	Anorexia
2.	Fatigue, general weakness and malaise	Malaise
3.	Altered level of consciousness, confusion, disorientation, meningeal signs, convulsion, seizures, and tremors	Neurologic disorders
4.	Nausea and vomiting	Nausea & vomiting
5.	Chest pain, difficulty in breathing and fluid in the lung cavity	Respiratory distress
6.	Conjunctivitis (red eyes), eye sensitivity and pain behind the eyes	Eyes symptoms
7	Muscle pain and side pain	Muscle pain
8.	Bleeding from any part of the body	Bleeding symptoms

### Definition of key variables

Hospital presentation time is the interval between the onset of LF symptoms and presentation at the hospital. Laboratory investigation commencement time is the interval between hospital presentation and sample collection. Diagnosis time from hospital presentation is the interval between hospital presentation and test result confirmation. Laboratory test turnaround time is the time from sample collection to test results release. The dependent variable was the case outcome, while symptoms, sociodemographic, and management factors were the independent variables. The analysis was guided by the assumption that LF survival may be influenced by sociodemographic characteristics, clinical severity, spatial distribution, health-seeking behaviour and health system management timelines.

### Geographical mapping of cases

The locations of confirmed cases were associated with their corresponding street addresses using Google Earth software [21]. Their locational coordinates were extracted based on their street addresses and not necessarily house addresses to protect their anonymity. The locations were plotted using ArcGIS Pro software. Spatial pattern analysis was conducted using geocoded case locations derived from the street addresses of the patients and was therefore based on case occurrence data rather than population-adjusted incidence rates. Point-based spatial statistics were used to examine the spatial concentration and clustering of reported cases across LGAs. Accordingly, the detected clusters represent spatial concentrations of reported cases, reflecting where cases were geographically concentrated rather than population-standardized disease risk. This methodological approach is appropriate for identifying spatial clustering patterns and informing targeted spatial surveillance, but does not constitute an incidence-based risk assessment. The results should therefore be interpreted in the context of case concentration analysis, rather than comparative incidence or risk across LGAs. For spatial auto-correlation analysis, point-level case locations were aggregated to LGA-level case counts. Spatial autocorrelation was evaluated using Global Moran’s I and Local Moran’s I at the LGA level. A first-order Queen contiguity-based spatial weights matrix (W) was employed, whereby LGAs sharing a boundary or vertex were considered neighbors. The weights matrix was row-standardized, ensuring that the sum of weights for each LGA equalled one. The same spatial weights matrix specification was applied consistently across both Global and Local Moran’s I analysis to ensure comparability of results.

### Ethical approval

Ethical approval was obtained from the Ebonyi State Ministry of Health Research and Ethics Committee and the University of Ibadan/University College Hospital Research Ethics Committee (UI/UCH REC; UI/EC/23/0394). This retrospective study involved secondary analysis of routinely collected medical records. All data were fully anonymised before access, and the requirement for informed consent was waived by the approving ethics committees.

### Statistical analysis

Microsoft Excel 2019, IBM SPSS Statistics version 26, and R version 4.3 were used for data analysis. Data were reviewed for completeness, duplicate entries, laboratory confirmation status, case outcome, and consistency of date variables used to compute time intervals. Records were included if they were laboratory-confirmed LF cases with documented outcome status. Categorical variables were summarized using frequencies and percentages, while continuous variables were summarized using medians and interquartile ranges. Missing values were described as unknown in descriptive analyses, while regression analyses were restricted to complete records for the variables included in each model. Case outcome, classified as alive or dead at hospital exit, was the primary outcome. Sociodemographic characteristics, clinical features, and management timeline variables were assessed as independent variables.

Chi-square or Fisher’s exact tests were used for bivariate associations, as appropriate. Variables with clinical relevance or significant bivariate association with case outcome were considered for multivariable analysis. Logistic regression was used to identify factors associated with survival status. Multicollinearity was assessed using the variance inflation factor, while model fit was assessed using the Hosmer-Lemeshow goodness-of-fit test. No major change in model estimates was observed after adjustment for clinically relevant variables.

Cox proportional hazards regression was used to assess factors associated with time to death/survival. For the survival analysis, time was measured from hospital presentation to recorded case outcome, with death treated as the event and discharge alive treated as censored. Records with missing management timeline data were excluded from the Cox model. The proportional hazards assumption was assessed using Schoenfeld residuals. The level of significance was set at 5%.

## Results

### Epi-curve of outbreak

**Fig 1:**
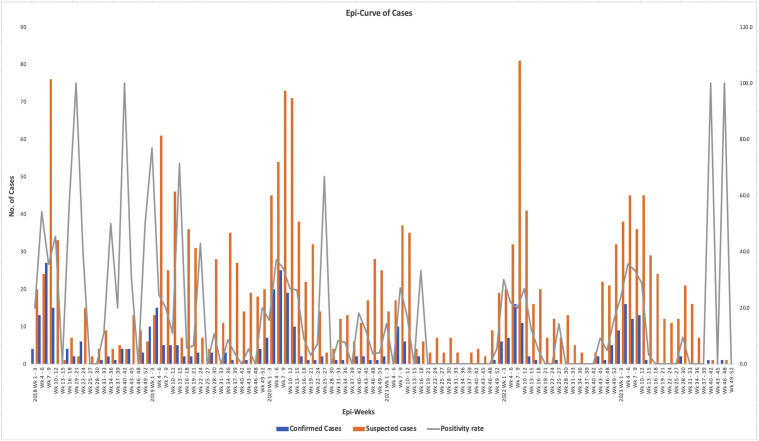
The weekly incidence of LF confirmed cases in Ebonyi State from January 2018 to December 2023.

A total of 368 LF cases were confirmed between January 2018 and December 2023. For the six years of study, the peaks of the LF incidence occurred between January and March. Notably, high peaks were observed during epi-weeks 4–6 in 2019 and 2023, and epi-weeks 7–9 in 2018, 2020, 2021, and 2022. The positivity rate curve showed no dramatic difference between the peak and non-peak periods (Fig 1).

### Outcome of cases

**Fig 2:**
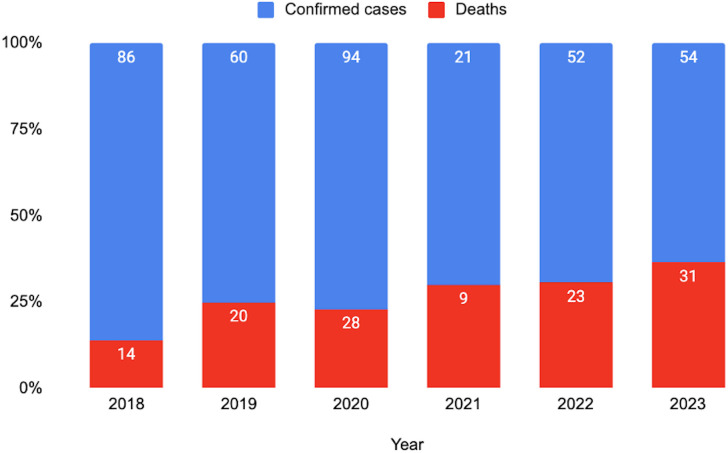
LF confirmed cases and deaths by year in Ebonyi State, 2018 - 2023.

Out of the 368 laboratory-confirmed LF cases, 125 died, resulting in a CFR of 33.97%. The highest CFR (57%) was recorded in 2023, and the lowest (16%) in 2018 (Fig 2).

#### Socio-demographic distribution of cases.

The median age was 32 (IQR 22 – 42) years. Among the cases, 52.7% were male. A large proportion of the cases, 144/298 (48.3%), had secondary education as their highest educational level, while 51/298 (17.1%) had primary education only. More than half (55.8%) of the cases were students, with 16.3% being farmers (Table 2).

**Table 2:** Socio-demographical characteristics of individuals with cases of LF (N = 368).

Variable	Frequency (n)	Percentage (%)	Alive n (%)	Dead n (%)	95% CI for Dead (%)
**Age Group**					
< 25 years	91	24.7	64 (70.3)	27 (29.7)	20.4 – 38.8
25 – 44 years	177	48.1	120 (67.8)	57 (32.2)	26.3 – 40.8
> 45 years	100	27.2	59 (59.0)	41 (41.0)	38.3 – 60.5
**Sex**					
Male	194	52.7	129 (66.5)	65 (33.5)	29.4 – 43.7
Female	174	47.3	114 (65.5)	60 (34.5)	28.4 – 43.1
**Education**					
No education	26	8.7	16 (62.4)	10 (37.6)	20.2 – 59.4
Primary	51	17.1	32 (62.7)	19 (37.3)	24.1 – 51.9
Secondary	144	48.3	84 (58.3)	60 (41.7)	33.5 – 50.2
Tertiary	77	25.8	53 (68.8)	24 (31.2)	21.1 – 42.7
Unknown	70	19.0†	58 (82.9)	12 (17.1)	9.2 – 28.0
**Occupation**					
Agricultural worker	60	16.3	30 (50.0)	30 (50.0)	38.5 – 65.0
Artisan	13	3.5	7 (53.8)	6 (46.2)	20.6 – 73.5
Businessman/woman	42	11.4	23 (54.8)	19 (45.2)	32.1 – 60.2
Civil servant	38	10.3	28 (73.7)	10 (26.3)	12.0 – 42.0
Clergy	2	0.5	2 (100.0)	0 (0.0)	–
Healthcare worker	27	7.3	21 (77.8)	6 (22.2)	6.0 – 42.0
Laborer	14	3.8	7 (50.0)	7 (50.0)	25.0 – 75.0
Pupil/Student	102	27.7	75 (73.5)	27 (26.5)	17.1 – 35.0
Unemployed	20	5.4	9 (45.0)	11 (55.0)	34.1 – 80.2
Unknown	50	13.6	41 (82.0)	9 (18.0)	8.7 – 40.2

+ Percentage calculated using the overall sample (N = 368); percentages for known education categories are based on participants with known education status (n = 298).

#### Documented clinical features.

**Fig 3:**
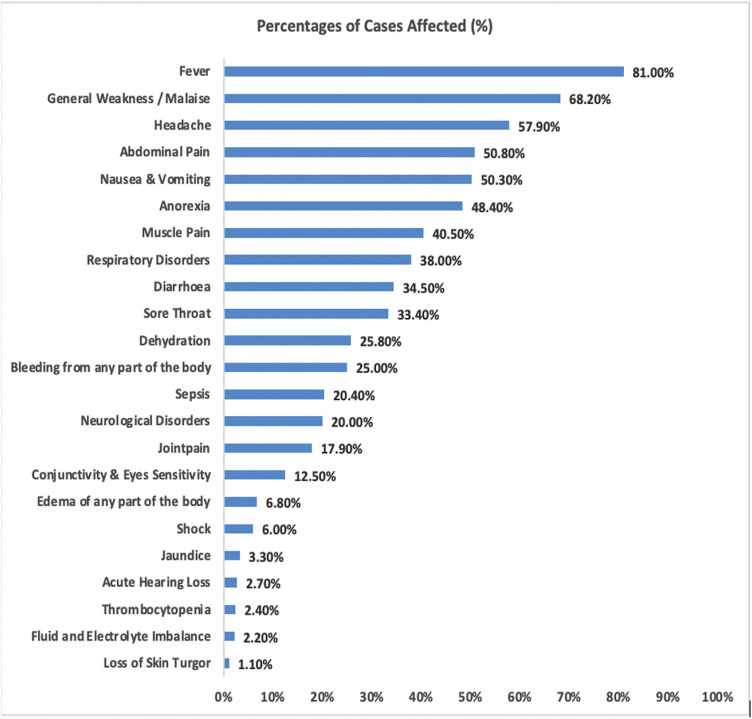
The common signs and symptoms observed among the cases.

Fever (81.0%), general weakness/malaise (68.2%), and headache (57.9%) were the most common clinical features observed among the cases. In contrast, thrombocytopenia (2.4%), fluid and electrolyte imbalance (2.2%), and loss of skin turgor (1.1%) were the least frequently documented clinical findings (Fig 3).

### Geographical distribution of cases

**Fig 4:**
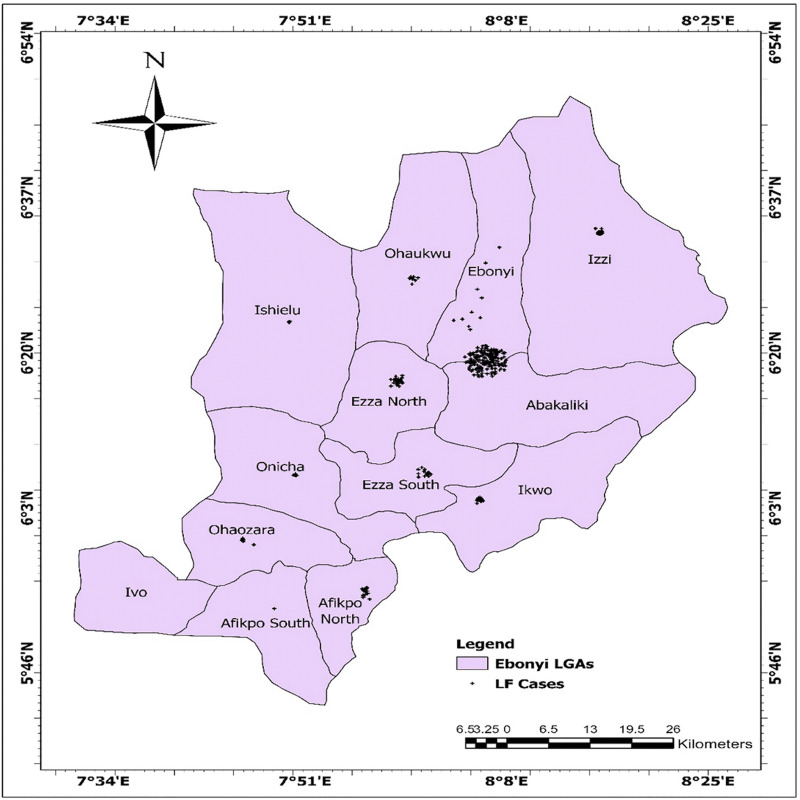
Geographical distribution of LF incidence across Ebonyi State from January 2018 to December 2023. Source: Department of Geography, University of Ibadan; https://Grid3.org. The administrative boundary used in the mapping of Lassa fever across LGAs in Ebonyi State was obtained from the Grid 3, licensed under Creative Commons Attribution 4.0 (CC BY 4.0). The link to LGA boundaries in Nigeria is: https://data.grid3.org/datasets/GRID3::grid3-nga-operational-lga-boundaries/about.

**Fig 5:**
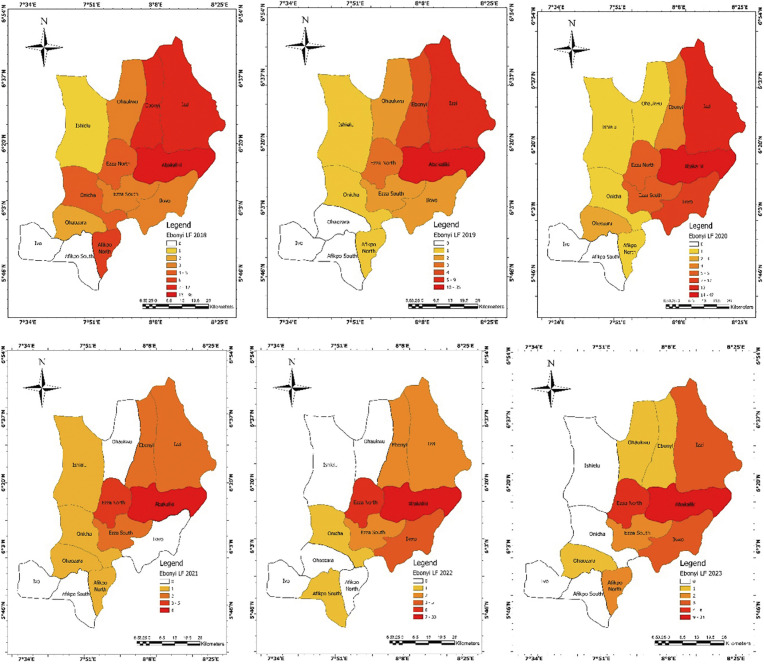
Thematic map showing the distribution of LF confirmed cases across Ebonyi State from January 2018 - December 2023. Source: Department of Geography, University of Ibadan; https://Grid3.org. The administrative boundary used in the thematic mapping of Lassa fever across LGAs in Ebonyi State was obtained from the Grid 3, licensed under Creative Commons Attribution 4.0 (CC BY 4.0). The link to LGA boundaries in Nigeria is: https://data.grid3.org/datasets/GRID3::grid3-nga-operational-lga-boundaries/about.

**Fig 6:**
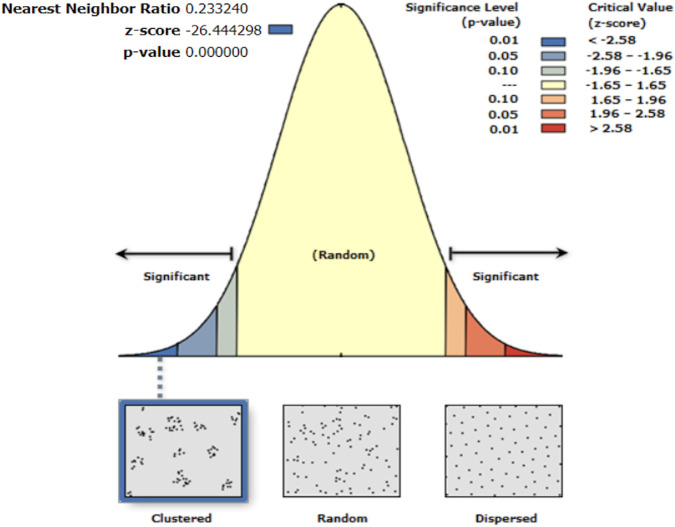
Nearest neighbor pattern distribution (2018-2023).

Of the 368 cases reported from the 13 LGAs of Ebonyi State, Abakaliki LGA reported 183 (49.7%), while Izzi LGA and Ezza North LGA reported 43 (11.7%) and 35 (9.5%), respectively. No cases were reported from Ivo LGA. Across the six years study period, the spatial distribution of reported LF were clustered (Nearest Neighbor Ratio: 0.2332, Z-Score: -26.44298, p-value: < 0.001) (Figs 4–6). Given that case locations were derived from street-level residential addresses, this result reflects clustering at the community or street level, where multiple cases may share similar or identical coordinates.

In contrast, the Global Moran’s I analysis assessed spatial clustering at the broader administrative scale by evaluating aggregated case counts across LGAs. For most years (2018, 2019, 2021, and 2023), Moran’s I values were near zero, with non-significant p-values (e.g., p > 0.05), indicating a random spatial distribution (Fig 6). There was no significant spatial pattern in these years, implying that LF cases were without an obvious geographic pattern. However, in 2020 and 2022, Moran’s I values were positive (0.261909 and 0.139280), with significant Z-scores and p-values (0.013171 and 0.020268), respectively, indicating clustered spatial distribution of Lassa fever cases (Table 3).

**Table 3:** Global Moran’s I result of Lassa Fever Cases in Ebonyi (2018-2023).

Year	Index	Z score	P value	Remark
2018	-0.0081282	0.482184	0.629675	Random
2019	-0.006151	0.707626	0.479178	Random
2020	0.261909	2.4791909	0.013171	Clustered
2021	-0.140603	-0.239770	0.810509	Random
2022	0.139280	2.321347	0.020268	Clustered
2023	0.025163	1.058802	0.289690	Random

**Fig 7:**
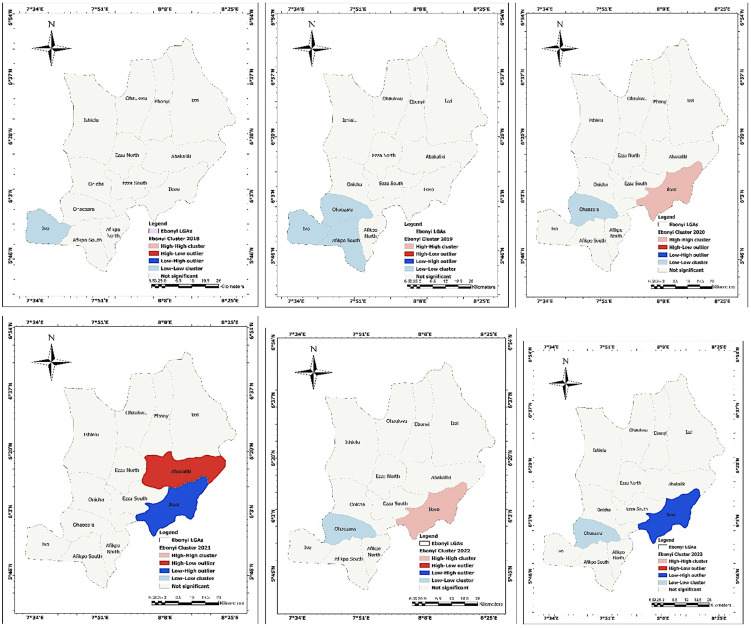
Local Spatial Clustering of Lassa fever Cases in Ebonyi State from 2018-2023. Source: Department of Geography, University of Ibadan; https://Grid3.org. The administrative boundary used in the thematic mapping of Lassa fever across LGAs in Ebonyi State was obtained from the Grid 3, licensed under Creative Commons Attribution 4.0 (CC BY 4.0). The link to LGA boundaries in Nigeria is: https://data.grid3.org/datasets/GRID3::grid3-nga-operational-lga-boundaries/about.

The Llysis results (Fig 7) depict the spatial clustering patterns of the LF cases.

In the early years of 2018 and 2019, the results mostly showed low-low clusters (light blue areas) in the southern part, which indicated LGAs with much lower-than-expected case counts and no obvious high-high clusters. This showed a low clustering of instances, with no place having exceptionally high concentrations. However, in 2020, there was an obvious change with the emergence of high-high clusters (red areas) in the northern and southern areas, indicating LGAs with much higher case clustering. Low-low clusters continued in the western parts, indicating a different pattern of high and low clustering in different LGAs.

In 2021 and 2022, the clustering pattern became more diverse, with some LGAs exhibiting emerging hot regions and others displaying cold spots. This variation implies varying spatial clustering, with particular LGAs having greater case concentrations. By 2023, a distinct low-high outlier formed in Ikwo LGA and a low-low cluster in Ohaozara LGA, indicating a low-level clustering of LF cases across the LGAs.

### Association between socio-demographic characteristics and clinical features with case outcome

There was no association between age, sex, education, and employment status with LF case outcome (p > 0.05). Abdominal pain, dehydration, diarrhoea, fever, sepsis, shock, sore throat, anorexia, malaise, neurologic disorders, nausea and vomiting, respiratory disorders, muscle pain, fluid and electrolyte disorders, and bleeding were associated with case outcome (p < 0.05) (S1 Table).

After adjusting for confounders, neurologic disorders, dehydration, bleeding, and nausea and vomiting were found to be the main predictors of LF case outcome. The odds of surviving LF were reduced by 64%, 59%, 48% and 47% in cases with neurologic disorders (OR: 0.36, 95% CI: 0.19 – 0.66), dehydration (OR: 0.41, 95% CI: 2.67 - 5.94), bleeding (OR: 0.52, 95% CI: 0.30 - 0.92), and nausea and vomiting (OR: 0.53, 95% CI: 0.32 - 0.89), respectively when compared with cases without the respective symptoms (S1 Table).

#### Probability of surviving LF infection in Ebonyi State with time.

**Fig 8:**
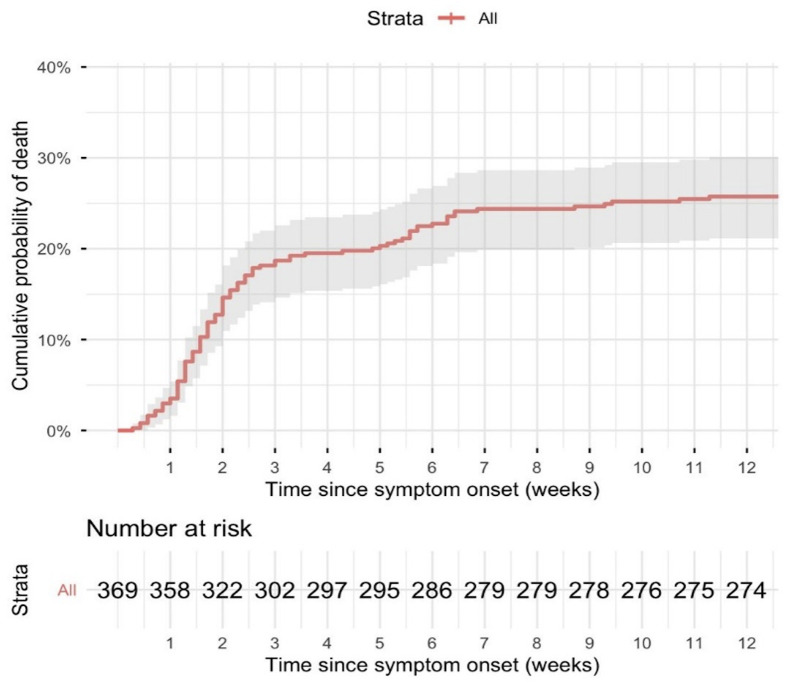
Probability of Surviving LF Infection in Ebonyi State from 2018–2023.

At LF onset, the cumulative probability of death is close to 0%, as expected. By around week 1, it has risen to about 5%. Between weeks 1–3, the curve rises more sharply, reaching roughly 18–19%. From week 3–5, the curve increases more slowly, reaching about 20%. There is another smaller rise around week 5–6, after which the curve begins to flatten. By week 7 onward, the cumulative probability of LF death remains fairly stable, ending at about 25–26% by week 12 (Fig 8).

### Health-seeking behaviour of cases

The median time from symptom onset to hospital presentation was 10 (IQR 7–18) days. The majority (60.6%) of the cases presented in the hospital after seven days from symptom onset (Table 4).

**Table 4:** Ebonyi State Management of LF Cases (N = 368).

Variable	Frequency (n)	Percentage (%)	Median Time in Days (IQR)
**Hospital Presentation Time (days)**			10 (7 - 18)
0 - 3	26	7.1	
4 - 7	48	13.0	
> 7	223	60.6	
Unavailable	71	19.3	
**Laboratory investigation commencement time (days)**			1 (0 - 4)
≤ 1	196	53.3	
2 - 4	47	12.8	
5 - 7	15	4.1	
> 7	39	10.6	
Unavailable	71	19.2	
**Laboratory test turnaround time (days)**			9 (1 - 17)
≤ 1	75	20.4	
2 - 4	30	8.2	
5 - 7	27	7.3	
> 7	165	44.8	
Unavailable	71	19.3	
**Diagnosis time from hospital presentation (days)**			12 (4 - 23)
≤ 1	56	15.2	
2 - 4	28	7.6	
5 - 7	26	7.1	
> 7	187	50.8	
Unavailable	71	19.3	

### Management of LF cases in Ebonyi

The median time from hospital presentation to investigation commencement was one day. Samples were collected later than one day for 46.7% of the cases. The median test turnaround time for LF was 9 days, and the LF diagnosis duration from time of hospital presentation was 12 days (Table 4). The hospital presentation and management timelines by year have been presented in S1 Table.

Among the variables included in the Cox proportional hazards model, age and diagnosis time from hospital presentation were significantly associated with the case outcome (p < 0.05). Specifically, patients aged >45 years had more than twice the hazard of dying compared with those aged <25 years (AHR: 2.20, 95% CI: 1.30–3.50). In contrast, patients whose diagnosis was made more than 3 days after hospital presentation had an 80% lower hazard of dying compared with those diagnosed within 3 days (AHR: 0.20, 95% CI: 0.10–0.40). Sex at birth, hospital presentation time, laboratory investigation commencement time, and laboratory turnaround time were not significantly associated with the hazard of dying (Table 5).

**Table 5:** Association of Management Variables with Outcome of Cases (without missing information) (N = 297).

Variable	Adjusted Hazard Ratio	95% CI	p-value
**Age**			
< 25	Ref	–	–
25–44	1.3	0.8 – 2.0	0.311
> 45	2.2	1.3 – 3.5	0.002**
**Sex at birth**			
Female	Ref	–	–
Male	1.0	0.7 – 1.4	0.967
**Hospital Presentation Time**			
≤ 7 days	Ref	–	–
> 7 days	0.7	0.5 – 1.2	0.217
**Laboratory investigation commencement time**			
≤ 1 day	Ref	–	–
> 1 day	1.2	0.8 – 1.8	0.440
**Lab turnaround time**			
≤ 1 day	Ref	–	–
> 1 day	0.5	0.3 – 1.0	0.052
**Diagnosis time from hospital presentation**			
≤ 3 days	Ref	–	–
> 3 days	0.2	0.1 – 0.4	< 0.001***

## Discussion

In this study, we provide a comprehensive analysis of the LF outbreak in Ebonyi State from 2018 to 2023, focusing on case outcomes, geographical distribution, health-seeking behaviour, and case management. The disease affects people of all ages; however, in this study, the median age of the cases was 32 years, which corresponds with an earlier study [22] in which most of the cases were aged between 22 and 42 years. Among the 368 confirmed cases, LF was almost equally spread across sexes, with a male-to-female ratio of 1.1:1.0. About half of the cases and deaths were recorded in Abakaliki LGA, the most developed and populous LGA in the state. Abakaliki LGA also hosts one of the state’s two main tertiary institutions, along with numerous secondary and primary schools, resulting in a large student population. The higher population density in Abakaliki LGA, driven by internal migration from less developed LGAs and compounded by the substantial student population, may partly explain the higher proportion of cases observed in this LGA. Notably, the absence of reported cases in Ivo LGA should be interpreted with caution, as it may reflect underreporting, differences in healthcare-seeking behaviour, or the use of alternative treatment pathways rather than a true absence of disease. Further investigation is needed to understand whether this pattern reflects lower transmission, limited case detection, or barriers to reporting from the LGA.

The observed spatial clustering in this study reflects areas with higher volumes of reported cases rather than true population-level incidence rates. For instance, the significant clustering observed in the Abakaliki LGA is heavily influenced by the presence of the AEFUTHA, which serves as the designated specialized treatment centre for Lassa fever (LF) in Ebonyi State. This institutional presence naturally drives a disproportionately high volume of direct self-presentations and inter-facility clinical referrals from across the state, thereby artificially inflating case clustering in this specific LGA. These findings underscore a critical structural gap in outbreak preparedness, highlighting the urgent need to establish decentralized, specialized care centres for viral hemorrhagic fevers across the region. Currently, patients must travel long distances to access specialized care. This geographic barrier likely contributes to the delayed hospital presentations observed in this study. Furthermore, it clarifies the inverse relationship between time-to-diagnosis and clinical outcome; individuals residing outside the Abakaliki LGA with mild symptoms are more likely to seek local, informal, or alternative treatments, delaying formal medical evaluation until clinical deterioration occurs.

The epi-curve showed that LF infection is highest during the dry season, as all the observed peaks fell between November and April. This aligns with a nationwide study that reported peak incidence of LF during the dry season and further confirms the findings of another study, which indicated that the LASV infection peaks during this period [23]. Although overall differences between peak and non‑peak periods were minimal, temporal trends in test positivity rate (TPR) reveal surveillance system maturation. In 2018–2019, peak‑month TPR exceeded 40%, suggesting a focus on severe cases testing. By 2020–2023, TPR stabilized at 10–20% despite a sharp rise in suspected case volume. This shift indicates expanded surveillance sensitivity, moving from detection of only severe presentations to capturing a broader range of suspected cases. Consequently, the higher number of confirmed cases in later years reflects both improved detection capacity and true transmission dynamics.

The overall CFR observed in this study was higher than the 26.2% CFR reported in another study from Ebonyi State covering January to March 2018 [18]. These rates are significantly higher than the WHO’s estimates of 1% for general LF cases and 15% for hospitalized cases [24]. The CFR in this study was also higher than the nationwide rate of 21% [25,26]. Although national LF surveillance reports show increasing case detection in recent years, the CFR in Ebonyi remained higher than national estimates, suggesting that the state-level pattern may reflect a greater concentration of severe hospitalised cases, delayed presentation, referral bias, and possible under-detection of mild community cases. The unusually high CFR of 57% recorded in 2023 is particularly alarming and may suggest that a larger proportion of cases reaching the treatment centre that year were already severely ill. This may have been influenced by late health-seeking behaviour, delayed referral, or delays in laboratory confirmation, although the available data do not allow us to determine the exact reasons for the peak in that year. The 2020–2023 period also coincided with COVID-19 and Mpox-related health system disruptions, which may have affected healthcare-seeking behaviour, referral pathways, laboratory workload and timeliness of LF response. Additionally, timely response management might have reduced the CFR observed in this study. For instance, the median duration for the LF test and diagnosis was nine and twelve days, respectively. These time periods are a bit late for the optimal benefit of Ribavirin which is usually more effective when started within six days of LF management [27].

According to WHO, clinical features like malaise, fever, headache, as well as vomiting and diarrhoea, which cause fluid and electrolyte imbalance, are early symptoms of LF, indicating a mild case, while neurologic disorders are symptoms of severe cases [24]. In this study, cases with clinical features relating to the neurologic system were less likely to survive. The odds of survival for those cases dropped by 53%. These findings align with another study in Ebonyi State, where neurologic features were associated with higher LF mortality [28]. Also, it corresponds with the WHO report that the probability of dying from LF significantly increases from mild to severe cases [24]. Notably, cases presenting general symptoms like fluid and electrolyte imbalance had significantly higher odds of surviving. Such cases might not have developed more serious complications like neurologic disorders, hence having better chances of surviving. These findings imply that LF cases presenting with neurologic symptoms should be given special attention in the management of LF outbreaks.

### Health-seeking tendency of the cases

The health-seeking behaviour of the cases was poor, as delayed hospital reporting among the cases was observed in this study. Half of the cases were reported to the hospital on the ninth day from the day of symptoms onset, and 68.2% reported after a week. Though this aligns with findings from Duvijnaud et al. (2021), where the median time of hospital presentation was eight days [22], it substantially differs from those of Chandra et al. (2021) and Ipadeola et al. (2023), where more than half of the cases presented in the hospital within six days of symptoms onset [29,30]. Because LF symptoms start like malaria, many people self-treat symptoms until they become overwhelmed by it, then seek hospital care. Research has also identified location, locality, and outcome of test presentation to be associated with hospital presentation time among LF cases [30]. Delayed presentation may also reflect socio-cultural and health system barriers, including low perceived risk of LF, reliance on self-medication or informal care, distance to appropriate facilities, referral delays, transport costs, and limited early suspicion of LF at lower levels of care. Early hospital presentation is important for the management of LF, as Ribavirin is more effective when initiated in a timely manner [27]. The delayed hospital reporting observed in this study might have negatively affected the chances of the cases benefiting optimally from treatment therapy. Hence, this might have contributed to the high CFR recorded. This highlights the need for intensive public health enlightenment that encourages early hospital reporting in Ebonyi State.

### Management of cases

The only treatment centre for LF in southeast Nigeria is the AEFUTHA [31]. It is one of the specialists’ facilities for LF and other hemorrhagic diseases in Nigeria. In AEFUTHA, the clinical management approach for suspected LF involves immediate isolation of the patient in an observation bay or holding area, notification of the responsible clinician and public health authorities, collection of samples for molecular detection of LASV by polymerase chain reaction (PCR) and severity assessment, and initiation of supportive care while awaiting confirmation. Standard initial care includes rehydration, provision of calories, monitoring of vital signs and oxygen saturation, antipyretics, antimalarials or antibiotics where indicated, oxygen therapy, anticonvulsants, antiemetics, antihypertensives, blood transfusion when required, and monitoring of urinary output. Confirmed cases are subsequently transferred to the treatment ward, while patients with negative results are decontaminated and transferred to routine medical care where appropriate. Intravenous Ribavirin remains the specific antiviral treatment for LF, and it is usually given for 10 days, with better outcomes expected when treatment is commenced early, particularly within six days of symptom onset. Severe cases require additional complication-directed care, including. renal, respiratory, hematologic, cardiovascular, nutritional, CNS, neonatal, and psychosocial support.

Prompt management is important for LF, but this can be challenging as early symptoms of LF appear like those of malaria. In this study, the timing of diagnostic management varied. Though the investigation commenced within twenty-four hours for most of the cases, it took an average of nine days to conduct the LF test and twelve days to confirm the LF diagnosis. The time taken to diagnose LF cases observed in this study was longer than that of Irrua Specialist Teaching Hospital, where LF diagnosis is made within twenty-four hours [32]. Interestingly, longer diagnosis time was associated with higher survival chances. This finding contradicts that of Cadmus et al. (2025) in Edo State, Nigeria, where diagnosis time was not associated with LF death [33]. This counterintuitive finding likely reflects disease severity, clinical prioritisation, and survival time bias rather than a true protective effect of delayed diagnosis. Since this study involved many hospitalized confirmed cases, the dataset may be biased towards severe presentations, consistent with WHO estimates that LF CFR is about 15% among hospitalised cases compared with 1% in non-hospitalised cases [24]. Severely ill patients may have been prioritised for faster investigation and diagnosis because of their clinical condition, but still had a higher mortality risk. In contrast, patients with milder infection may have survived long enough to receive diagnosis later, making longer diagnosis time appear associated with survival. Nevertheless, timely diagnosis remains critical in LF management because Ribavirin is most effective when initiated early [27].

An important trend observed over the study period is the sustained rise in CFR, which reached 56.4% in 2023. This increase likely reflects multiple factors, including delays in health‑seeking behaviour and diagnostic turnaround, but may also point to broader challenges such as post‑pandemic shifts in healthcare access, barriers to early presentation, or evolving clinical profiles. The rising CFR underscores a pressing public health concern for Ebonyi State and highlights the need for further investigation into the drivers of LF mortality.

## Strengths and limitations of the study

- We described in-depth the epidemiology, geographical distribution, and health-seeking behaviour of LF in Ebonyi State from January 2018 to December 2023.
- Since this study was a secondary analysis, there was no access to the hospital case file and test protocols; hence, the exact cause of death for the cases and the details of the PCR test assays/platforms/protocol could not be provided.
- The years 2020–2023 presented here had major global outbreaks like the COVID-19 pandemic and Mpox outbreak, which would have affected the timeliness and effectiveness of the response to LF, an endemic disease.
- This study is subject to the MAUP, as point-level LF cases were aggregated to the LGA level. Such aggregation may influence the observed spatial patterns and should be taken into account when interpreting the findings. Data privacy constraints and routine reporting practices limit the use of finer geographic units.
- Excluding suspected, pending, negative, and “not a case” records may have underestimated the broader suspected LF burden and does not fully rule out misclassification from false-negative results, incomplete testing, or surveillance classification errors.
- As this study used routine surveillance data, the findings may be affected by underreporting and changes in surveillance sensitivity or testing capacity over time. Although the NCDC LF case definitions remained consistent during the study period, improvements in case detection and testing may have influenced temporal trends, confirmed case counts and CFR estimates.
- Therefore, the observed associations should be interpreted cautiously as markers of clinical severity and possible care pathway delays, rather than direct independent predictors of outcome.

## Conclusion

This study shows that LF in Ebonyi State was associated with a high CFR, with the largest concentration of reported cases and deaths occurring in Abakaliki LGA. Clinical features suggestive of severe disease, particularly neurologic disorders, dehydration, bleeding, and nausea/vomiting, were associated with reduced survival. Although delayed hospital presentation was common, it was not significantly associated with case outcome in the survival model. The long laboratory turnaround and diagnosis timelines, however, point to important operational gaps in case management that require attention.

## Recommendation

In the management of LF outbreaks in Ebonyi State, suspected cases presenting with neurologic symptoms and other signs of severe disease should be prioritised for urgent clinical assessment and care. The diagnostic management algorithm should be reviewed to reduce delays in sample transport, laboratory processing, and communication of test results. Early triage and referral systems should be strengthened, particularly at lower-level facilities, to support timely identification and transfer of suspected cases to the designated treatment centre. Community risk communication should focus on early recognition of persistent fever or malaria-like illness that does not respond to treatment and encourage prompt use of appropriate health facilities. Future studies should use finer geographic units or multi-scale spatial approaches to improve the interpretation of spatial patterns.

## Supporting information

S1 Table Presentation and management timelines by year. (DOCX)

S1 Data Study data. (XLSX)
