# Supplementary material for: Temporal trends and epidemiological patterns of Lassa fever outbreaks in Ebonyi State, Nigeria: A retrospective study (2018–2023)
Source: PLOS Glob Public Health. 2026 Jul 28;6(7):e0006871. doi: 10.1371/journal.pgph.0006871 (PMC13411902; doi:10.1371/journal.pgph.0006871)
Supplement: S1 Table — (DOCX) [file pgph.0006871.s001.docx]

**S1 Table 1: Presentation and Management Timelines by Year**

| **Year** | **Presentation time, median (IQR)** | **Investigation commencement time, median (IQR)** | **Test duration, median (IQR)** | **Diagnosis time, median (IQR)** |
| --- | --- | --- | --- | --- |
| 2018 | 26.0 (16.5–35.3) | 0.0 (0.0–13.3) | 12.5 (4.0–21.0) | 19.5 (7.8–37.3) |
| 2019 | 9.0 (7.0–14.0) | 3.0 (1.0–5.0) | 10.0 (2.0–19.0) | 14.0 (5.0–23.0) |
| 2020 | 8.0 (4.0–14.0) | 1.0 (1.0–5.0) | 10.0 (3.3–17.8) | 14.0 (8.0–25.5) |
| 2021 | 13.0 (9.0–18.0) | 0.0 (0.0–1.0) | 6.0 (1.0–12.0) | 6.0 (2.0–13.0) |
| 2022 | 9.0 (7.0–14.0) | 0.0 (0.0–1.0) | 9.0 (1.0–13.0) | 9.0 (1.0–14.0) |
| 2023 | 11.0 (8.8–15.3) | 0.0 (0.0–0.0) | 6.0 (0.0–9.3) | 6.5 (0.0–10.0) |
